# Effect of skin infiltration with ropivacaine on postoperative pain in patients undergoing craniotomy

**DOI:** 10.1186/s40064-016-2856-3

**Published:** 2016-07-26

**Authors:** Hongyu Zhou, Mengchan Ou, Yaoxin Yang, Qian Ruan, Yan Pan, Yu Li

**Affiliations:** Department of Anesthesiology, West China Hospital, Sichuan University, Chengdu, Sichuan People’s Republic of China

**Keywords:** Craniotomy, Scalp infiltration, Postoperative pain, Ropivacaine

## Abstract

**Background:**

Local anesthetic infiltration has been used to manage postoperative pain in various surgeries. The present study was aimed to investigate the effect of skin infiltration with 0.5 % ropivacaine on postoperative pain in patients undergoing craniotomy.

**Methods:**

One hundred and six patients with ASA I/II scheduled to undergo elective craniotomy were enrolled during March to November in 2015 in this prospective, randomized, placebo-controlled, double-blind study. After the anesthesia induction, skin along the incision was infiltrated with 0.5 % ropicavaine (group R, n = 53) or 0.9 % normal saline (group C, n = 53), respectively. Morphine was used as rescue analgesic postoperatively. Morphine consumption during the first 24 postoperative hours was recorded as the primary outcome, and the time to first rescue requirement was also recorded. Pain was assessed at 2, 4, 8, 24 h, 7 days, 3 months after surgery by visual analog scale (VAS). Heart rate and mean arterial pressure were recorded before anesthesia induction (T1), after anesthesia induction (T2), after scalp infiltration (T3), during skull drilling (T4), mater cutting (T5) and skin closure (T6).

**Results:**

Morphine consumption during the first 24 postoperative hours was significantly higher in group C than in group R (13.36 [6.5, 20] vs. 6.3 [0, 10] mg, *P* < 0.05). The first time of patients needed rescue analgesic was prolonged in group R as compared with group C (6.16 [3.4, 8.0] vs. 3.87 [2.3, 4] h, *P* < 0.05). Postoperative VAS and hemodynamic signs during the first 24 h showed no significant difference in two groups. The incidence of persistent pain on 7 days and 3 months postoperatively had no significant differences between two groups. Besides one patient (2 %) enduring moderate pain (VAS 4–7) in group C, the number of patients suffering from mild pain (VAS 1–3) was 17 (33.3 %) in group R and 17 (34 %) in group C 3 months after surgery.

**Conclusion:**

The results suggest 0.5 % ropivacaine scalp infiltration before skin incision has favorable analgesic effect in reducing morphine consumption and prolong the time of first rescue analgesic requirement after surgery.

*Trial registration* Chinese Clinical Trial Registry (http://www.chictr.org.cn/) registration number: ChiCTR-IPR-14005717

## Background

Pain following craniotomy has been neglected for decades because of the presumed absence of nociceptors in the brain parenchyma, and innervation virtually limited to the meninges, pericranial muscles and fascia (De Benedittis et al. 1996). In recent studies, patients following craniotomy revealed that they experience more than minimal pain. Mordhorst et al. (2010) reported that up to 55 % of patients had moderate or severe postoperative pain in the first 24 h after undergoing a craniotomy. The incidence of chronic pain lasting more than 3 months post-craniotomy was 28.4 % postoperatively. Additionally, 14.2 % of the patients reported slight chronic pain and 18.5 % reported that their daily lives were heavily affected by pain (Ryzenman et al. 2005).

The significant pain resulting from scalp incision is a strong nociceptive stimulus that can provoke abrupt changes in sympathetic activity and haemodynamics (Hillman et al. 1987). Acute hypertension is deleterious for neurosurgical patients because it may translate into increasing intracranial pressure and increased risk of cerebral aneurysm rupture. This effect can result in haemorrhage, cerebral hernia or pulmonary oedema (Basali et al. 2000), all of which are fatal complications. Research has shown that sufficient analgesia can prevent common postoperative side effects, enhance patient comfort and facilitate the recovery process (Lux et al. 2011; Taylor et al. 2013). Therefore, effective perioperative pain management is highly demanded to prevent the acute hypertension-related morbidity in neurosurgical patients.

Local anaesthetic infiltration of the scalp has been proposed to lessen intraoperative haemodynamic responses to craniotomy (Bloomfield et al. 1998). However, the efficacy of local anaesthetic wound infiltration for the treatment of acute pain after neurosurgery is controversial (Song et al. 2015; Law-Koune et al. 2005) probably because of the heterogeneity of studies. Further, according to previous studies (Bisgaard et al. 2005), the intensity of acute pain postoperatively may be associated with a high incidence of chronic pain. Despite the aforementioned high prevalence of chronic pain post-craniotomy (Mordhorst et al. 2010), no authoritative therapeutic guidelines on chronic post-craniotomy headache are available. This makes pain management particularly difficult. Therefore, the aim of this prospective, randomised study was to explore the hypothesis that the preemptive skin infiltration with 0.5 % ropivacaine in patients undergoing craniotomy is effective for postoperative acute pain management and might contribute to reducing the incidence of chronic pain.

## Methods

The Ethical Committee at our institution approved this prospective, randomised, double-blinded study. Ethical approval for this study (Ethical Committee No. 213) was provided by the Ethical Committee of West China Hospital, Sichuan, China (Prof Li) on 10 December 2014. The written informed consent was obtained from all patients preoperatively. We also registered the study protocol in the Chinese Clinical Trial Registry (registration number: ChiCTR-IPR-14005717). One hundred and fifty-four patients aged between 18 and 70 years with American Society of Anesthesiologists (ASA) physical status I/II who were scheduled for elective craniotomy under general anaesthesia were recruited in this study.

The exclusion criteria included inability to understand and use the visual analogue scale (VAS), patients undergoing intervertebral anaesthesia and/or epidural analgesia, patients managed with postoperative patient-controlled epidural analgesia or postoperative intravenous analgesia, inability to communicate because of impaired consciousness (Glasgow Coma Score less than 15), proven or suspected allergy to local anaesthetics, a previous scalp incision, and patients treated with opioids for more than 14 days in the last 3 months or nonopioid analgesics at a frequency greater than 5 times per week.

The day before the surgery, all enrolled patients received explanations as to the usage of a 10-cm VAS (0 = no pain, 10 = the worst pain) by the investigator. No premedication was administered preoperatively. The randomised group assignment was performed with a computer-generated random number table. The randomisation information was concealed by using opaque envelopes. In the ward, after undergoing a preoperative interview, patients considered eligible received a unique random code and were randomly assigned into either the ropivacaine group (group R) or the placebo group (group C). On the day of surgery, according to the random code of the patient, an anaesthetic nurse prepared the solution of normal saline or ropivacaine. All solutions were prepared in identical syringes. The random code, patient’s information and group name were enclosed in the sealed opaque envelope. The anaesthetists who performed the anaesthesia and recorded the intraoperative data, the neurosurgeons who performed the scalp infiltration, and patients were all blinded to the group assignment. The envelope was opened only if emergency unblinding was required. All the follow-up procedures were conducted by another nurse who was also blind to the treatment group assignment.

The electrocardiogram, noninvasive blood pressure and pulse oximetry were monitored continuously. Anaesthesia was induced with sufentanil 0.2–0.3 µg/kg, midazolam 0.05 mg/kg, and propofol 2 mg/kg. Tracheal intubation was facilitated by rocuronium 0.7 mg/kg. After the induction of general anaesthesia, the radial artery was cannulated and connected to a transducer placed at the midaxillary level. Propofol (4–12 mg/kg/h), remifentanil (0.1–0.2 mg/kg/min) and 1.0 minimum alveolar concentration of sevoflurane mixed with oxygen (50 %) and air (50 %) were used for maintenance of anaesthesia and adjusted to maintain the mean arterial pressure (MAP) within 60 and 80 mmHg. Ventilation was mechanically controlled to achieve an end-tidal CO_2_ (ETCO_2_) of 30–35 mmHg.

The neurosurgeon infiltrated the scalp along the incision site 5 min before the surgery. Group R received 10 ml of 0.5 % ropivacaine while group C received an equal volume of 0.9 % normal saline solution.

Heart rate (HR) and blood pressure (BP) were measured before anaesthesia induction (T1, baseline value), after anaesthesia induction (T2), after scalp infiltration (T3), during skull drilling (T4), dura mater cutting (T5) and skin closure (T6). The total consumption of sufentanil and remifentanil during the operation were recorded. The BP and HR were maintained within the 20 % from the preoperative baseline value through the surgery via adjusting the anaesthetic dosage or administration of vasoactive agents such as nicardipine.

Postoperative VAS scores, postoperative nausea and vomiting (PONV) scores, sedation scores, HR and BP were recorded at 2, 4, 8, and 24 h postoperatively. Morphine was used as rescue analgesic during the first 24 h postoperatively. If the measured VAS was >4 or patients requested analgesia, the rescue analgesia was administered by a nurse as an intravenous injection of morphine 5 mg. Intravenous morphine 2.5 mg was given repeatedly at a minimal interval of 10 min if needed until VAS was <4.

The primary outcome of this study was morphine consumption during the first 24 h postoperatively. The consumed dosage of morphine and the time to first rescue requirement were recorded carefully. The patients’ satisfaction with postoperative analgesia was recorded at 24 and 48 h postoperatively. The VAS at 7 days and 3 months postoperatively were assessed. Patients whose VAS was ≥1 at 3 months postoperatively were diagnosed with chronic postoperative pain. Adverse events (AEs) and serious adverse events (SAEs) were recorded throughout the study period. An AE was defined as any untoward medical occurrence. An SAE included death, immediately life-threatening conditions, coma, in-patient hospitalisation or prolongation of existing hospitalisation.

### Statistical analysis

All statistical analyses were performed with IBM SPSS statistics 17.0 software (IBM Corporation, Armonk, New York). Normally distributed continuous variables were present as means ± stand deviation, and Student’s t test was used to determine the significance of the difference between the two independent groups. Repeated measured continuous variables were analysed with repeated-measures analysis of variance to detect differences. Non-normally distributed continuous variables were presented as median (quartile range) and were analysed with Mann–Whitney U test or Kruskal–Wallis test. Dichotomous variables were analysed with Chi square tests. Difference was considered statistically significant when the *P* value was <0.05.

## Results

**Fig. 1:**
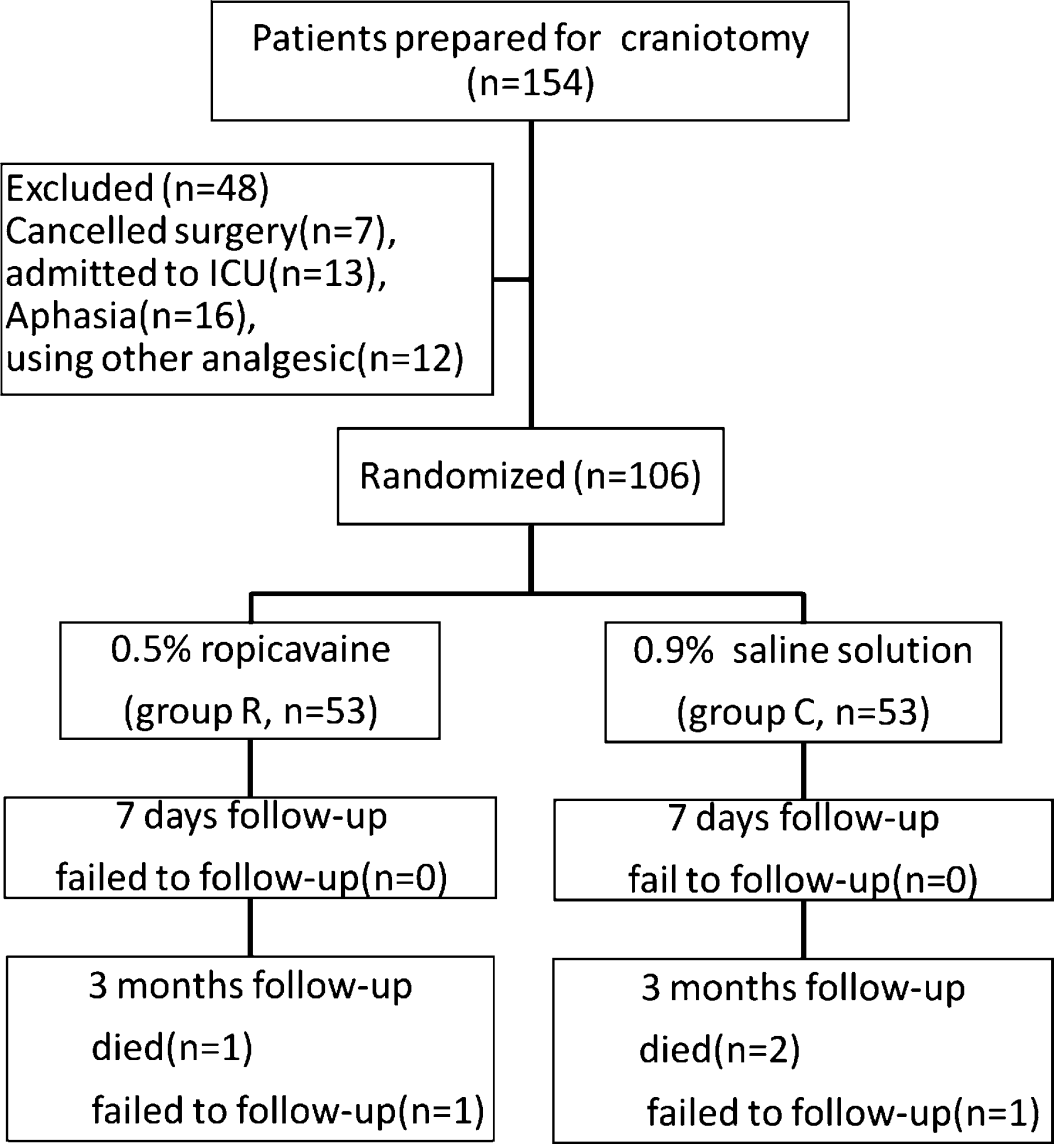
Flow of patients through testing and follow-up

One hundred and fifty-four patients were enrolled. Of these, the surgeries of seven patients were cancelled after randomisation, 13 patients were admitted to ICU for ventilation, 16 patients were aphasic or were incapable of communicating, and 12 patients used other analgesics rather than morphine for postoperative analgesia. Thus, 106 patients were analysed (53 in group R and 53 in group C). No patient was lost to follow-up during the first 7 days postoperatively. Results for 3 months postoperatively are reported for 50 patients in group C and 51 in group R because five additional patients were excluded from the study (Fig. 1): three patients died (1 patient in group R, 2 patients in group C) and two patients were lost to follow-up (1 patient in each group). There were no significant differences with respect to patient characteristics and duration of surgery between the two groups (Table 1).

**Table 1:** The characteristic of patients undergoing craniotomy (n = 106)

	Group R (n = 53)	Group C (n = 53)	*P* values
Age (year)	48.26 ± 11.99	49.00 ± 13.28	0.765
Sex			
Female	23	26	0.559
Male	30	27	
BMI (kg/m^2^)	22.08 ± 2.36	21.51 ± 2.01	0.187
ASA			
I	9	8	0.791
II	44	45	

**Fig. 2:**
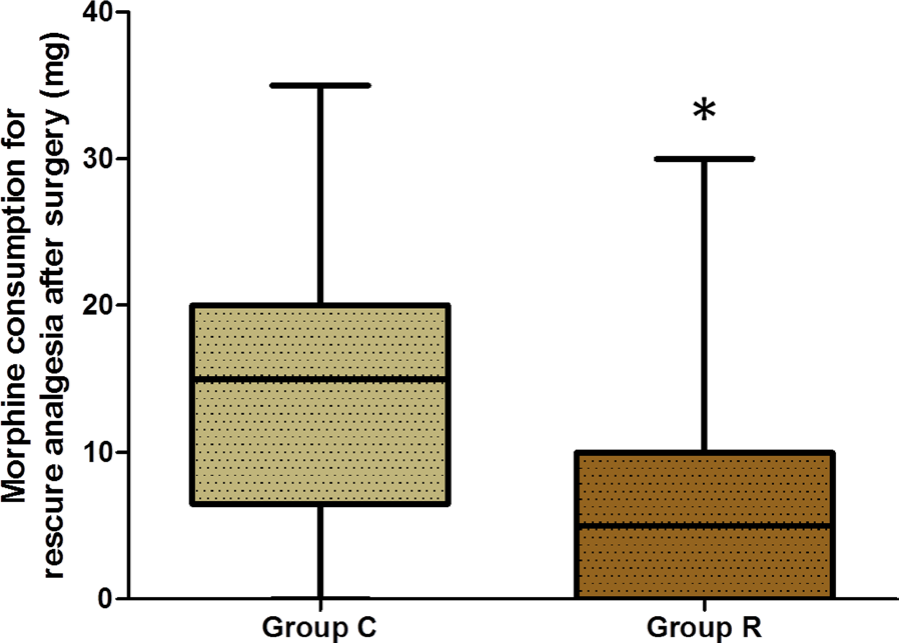
Comparison of morphine consumption during the first 24 postoperative hours. *Group C* control group, *Group R* ropivacaine group. *P < 0.05 versus control group

**Fig. 3:**
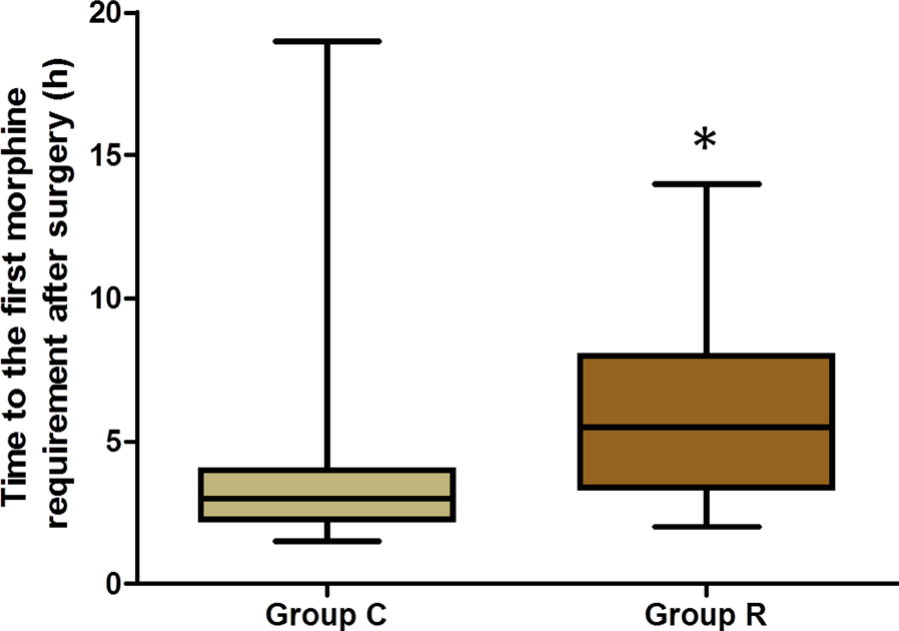
Comparison of the first time patients requested rescue analgesia. *Group C* control group, *Group R* ropivacaine group. *P < 0.05 versus control group

**Fig. 4:**
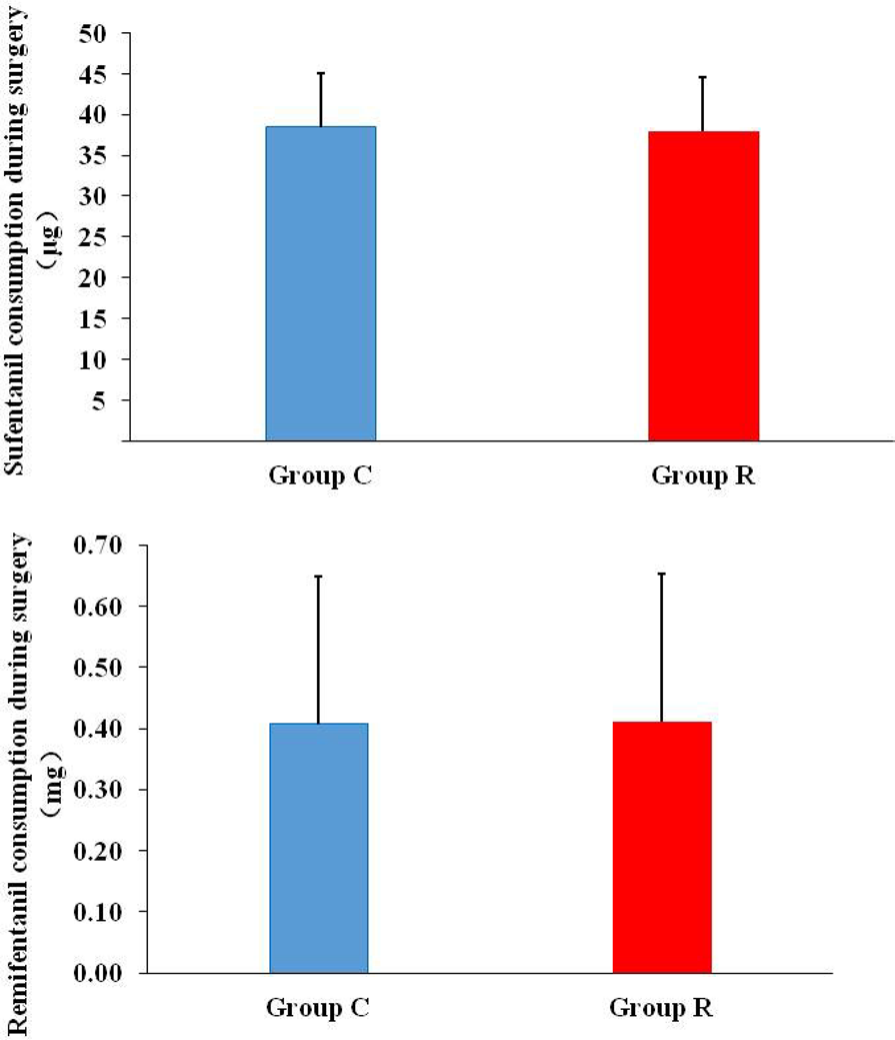
Comparison of sufentanil and remifentanil consumptions during the surgery. *Group C* control group, *Group R* ropivacaine group

Morphine consumption during the first 24 postoperative hours was significantly higher in group C than in group R (13.36 [6.5, 20] vs. 6.3 [0, 10] mg, *P* < 0.05; Fig. 2). The first time patients requested rescue analgesia was prolonged in group R as compared with group C (6.16 [3.4, 8.0] vs. 3.87 [2.3, 4] h, *P* < 0.05; Fig. 3). The consumed dosage of sufentanil and remifentanil during the surgery was lower in group R than in group C, but the difference was not significant (*P* > 0.05; Fig. 4).

**Fig. 5:**
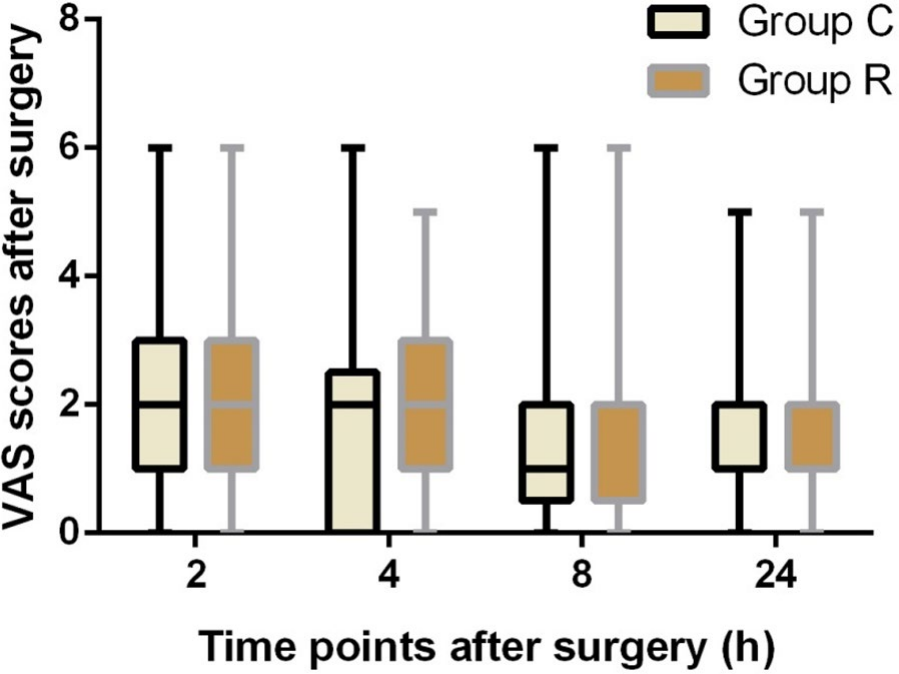
Comparison of VAS scores in both the groups after surgery. *Group C* control group, *Group R* ropivacaine group

The assessment of postoperative VAS at several time points during the first 24 h postoperatively did not reveal any significant differences between groups. The incidence of pain was 77.4 % in group R and 79.2 % in group C at the first 2 h postoperatively. Six (11.3 %) patients suffered from moderate or severe pain in group R and 11 (20.7 %) in group C at the 2 h postoperatively, but the difference was not significant (*P* > 0.05; Fig. 5). The prevalence of postoperative pain at 7 days postoperatively and the incidence of chronic postoperative pain at 3 months were not significantly different between groups (Table 2). Aside from one patient (2 %) who presented moderate pain in group C, 17 (33.3 %) patients in group R and 17 (34 %) in group C presented mild pain at 3 months postoperatively (Table 3).

**Table 2:** Prevalence of pain at 1 week after surgery and 3 months after surgery

	1 week after surgery	3 months after surgery
Group C	27/53	18/50
Group R	26/53	17/51

**Table 3:** The intensity of pain in 3 months postoperatively assessed by VAS

	No pain	Mild pain	Moderate pain
Group C	34/51	17/51	0
Group R	32/50	17/50	1/50

No pain = 0, mild pain = 1–3, moderate pain = 4–7, severe pain = 8–10

**Fig. 6:**
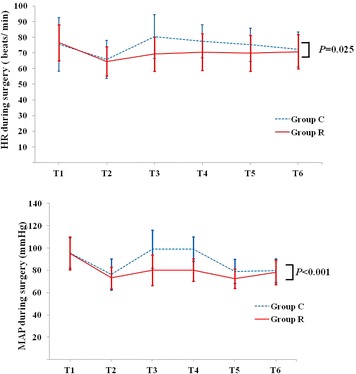
Comparison of HR and MAP changes during surgery. *T1* before anaesthesia induction, *T2* after anaesthesia induction, *T3* after scalp infiltration, *T4* during skull drilling, *T5* dura mater cutting, *T6* skin closure. *Group C* control group, *Group R* ropivacaine group

**Fig. 7:**
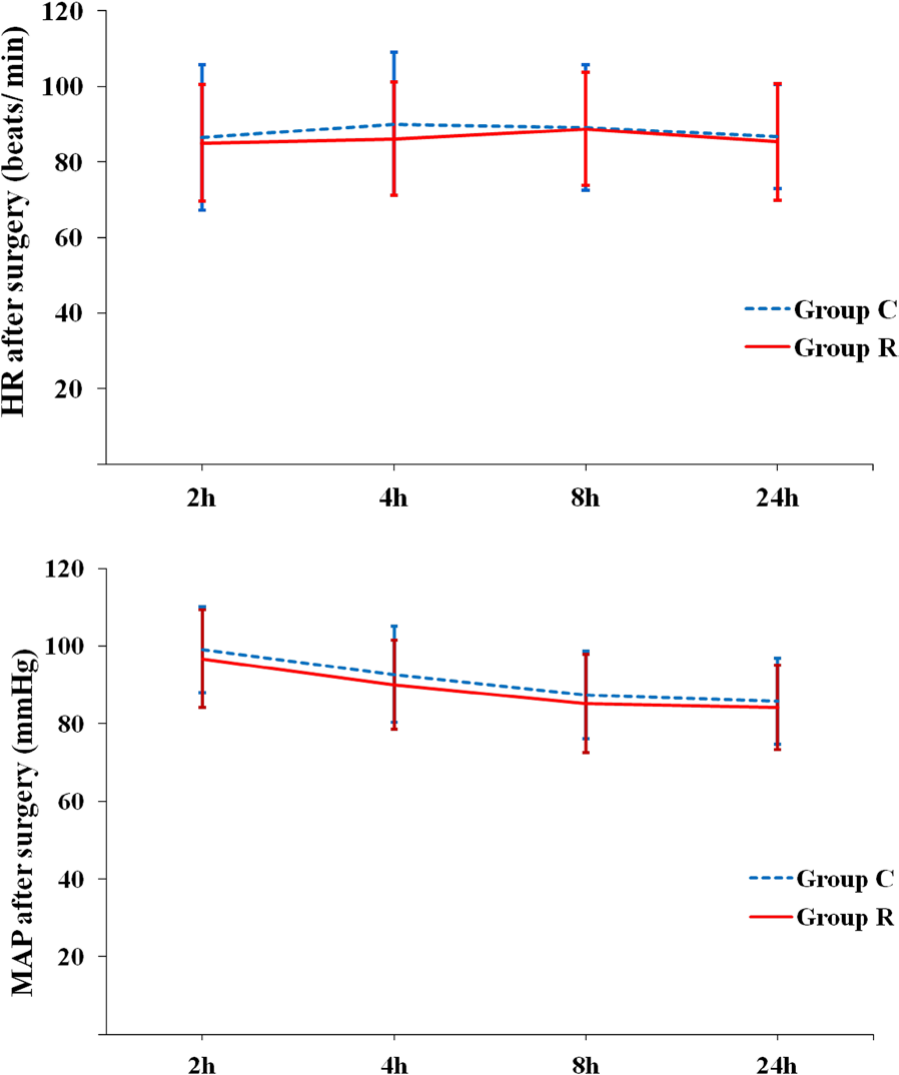
Comparison of HR and MAP changes after surgery. *Group C* control group, *Group R* ropivacaine group

There were no significant differences between groups for HR and MAP at each intraoperative time point, except for T3 and T4. At the time points of T3 and T4, the HR and MAP in group C were significantly higher than in group R. There were significant differences in HR and MAP before and after the skin incision as shown by within group analysis. However, the changes in HR and MAP between the time points, T2 and T3, were significantly lower in group R than that in group C (*P* < 0.05; Fig. 6). The HR and MAP at every postoperative time point were not significantly different between groups (Fig. 7).

**Fig. 8:**
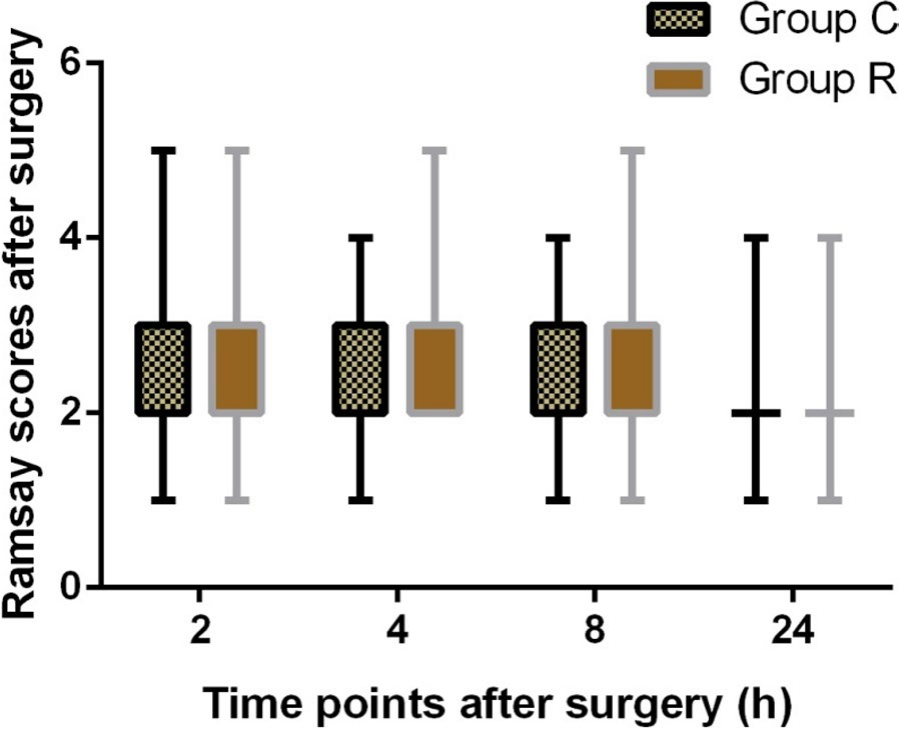
Comparison of postoperative Ramsay scores in both the groups after surgery. *Group C* control group, *Group R* ropivacaine group

In both groups, the postoperative Ramsay scores were not significantly different at any time point (*P* > 0.05; Fig. 8). The incidence of PONV at each time point, except for the first 8 h postoperatively (10 vs. 3), was not significantly different between groups (Table 4). No patient experienced respiratory depression (defined as a respiratory rate <10 breaths per minute or SpO_2_ < 90 %). The length of hospital stay was not significantly different between groups.

**Table 4:** Incidence of postoperative nausea and vomiting

	2 h	4 h	8 h	24 h
Group C	6/53	5/53	10/53	10/53
Group R	5/53	5/53	3/53*	10/53

* *P* < 0.05 versus group C

## Discussion

This prospective, randomised, double-blinded, placebo-controlled study showed that the scalp infiltration performed preoperatively with 0.5 % ropivacaine decreased the morphine consumption for rescue analgesia within 24 h postoperatively as well as the incidence of PONV at 8 h. However, this intervention had no effect on chronic postoperative pain at 3 months, and did not increase the incidence of postoperative AEs. In terms of optimal postoperative pain management regimen, in the present study, VAS scores at different time points were not significantly different between groups. However, the reduced morphine consumption during the first 24 h postoperatively and the prolonged time of first rescue analgesic requirement were evidence of the beneficial effect of the ropivacaine scalp infiltration for pain management in craniotomy patients.

Ropivacaine, the local anaesthetic used in our study, has a biphasic vascular effect. It is reported that 0.5 % ropivacaine can contract the blood vessels, which is the main function of epinephrine (Hansen 2004). Epinephrine was not used in our study because of the haemodynamic changes it causes, especially in patients with aneurysms (Yang et al. 2007).

Considering the serious complications caused by headaches postoperatively, it is important to select an effective analgesic to alleviate postoperative pain. No ideal analgesic has been described for use in patients undergoing craniotomy. Despite the lack of clinical evidence related to craniotomy, nonopioid analgesics such as nonsteroidal anti-inflammatory drugs, and opioids are widely used to relieve postoperative pain (Gottschalk and Yaster 2009; Molnar et al. 2014). Opioid analgesics are the main intervention for pain control in patients. Moore et al. (2015) reported that dexketoprofen trometamol 25 mg combined with tramadol hydrochloride 75 mg had favorable analgesic effect on moderate to severe pain. Morphine has been found to be the most effective analgesic among these agents, it is the gold standard of moderate and severe pain management (Gottschalk and Yaster 2009).

Retrospective and prospective studies provide conflicting results regarding scalp infiltration with local anaesthesia, such as diverse changes of perioperative haemodynamics, VAS scores, and morphine consumption. Pre-incision scalp infiltration was studied by Biswas and Bithal (2003): they concluded that the scalp infiltration may delay the requirement of the first analgesic dose. They assumed that in the bupivacaine group, the duration of skin hypoesthesia produced by 0.25 % bupivacaine (Swerdlow and Jones 1970) was longer than the duration of surgery time, which contributed to the apparently delayed requirement of the first analgesic dose. In our study, the time to first rescue analgesic requirement was prolonged in group R compared with group C. However, we found that despite the similarity of postoperative VAS scores in groups R and C, morphine consumption during the first 24 postoperative hours was significantly higher in group C than in group R. Additionally, in some cases, morphine was used before the time points of VAS assessment, which may have caused discordance between morphine consumption and the VAS scores obtained.

In contrast, Law-Koune et al. (2005) assessed the benefit of scalp infiltration on acute postoperative pain and found that there was a transient effect with ropivacaine or bupivacaine, which lasted only 1 or 2 h. Batoz et al. (2009) considered that the small sample size and the difficulty of obtaining a reliable pain evaluation by VAS may also be accountable for ineffective analgesia. Furthermore, pain was referred to as predominantly superficial in 86 % of the patients, suggesting somatic rather than visceral origin and possibly mainly involving pericranial muscles and soft tissues (De Benedittis et al. 1996). Nevertheless, in our study, we only infiltrated the subcutaneous tissue at the site of the scalp incision, which may result in unsatisfactory analgesia.

Opioids can increase the prevalence of nausea and vomiting. In our study, the incidence of PONV in group R was significantly reduced at 8 h compared with group C. It is reported that postoperative pain and the opioids used are associated with PONV (Kenny 1994). PONV can cause tension on the suture lines, hypertension, increased bleeding under skin flaps, among other complications (Camu et al. 1992). Therefore, we consider that the reduction of opioid consumption in the present study contributed to fewer opioid-related AEs.

As far as we know, only a few studies evaluated chronic postoperative headache. In particular, whether chronic pain can be relieved by pre-incisional scalp infiltration with local anaesthetics has been assessed rarely. In our study, chronic pain assessed by VAS at 3 months postoperatively was not significantly different between groups. Multiple reasons may account for this phenomenon. First, as related studies indicated, acute pain can progress to chronic pain. The origin of most acute pain is the locally damaged tissue and inflammatory response (Kaufman et al. 2005). It is the development of peripheral and central sensitisation—the plasticity of the nervous system after an injury—that leads to a reduced pain threshold, amplification of pain response, and spread of pain to non-injured areas (Katz et al. 2003; Mifflin and Kerr 2014). Neil and Bannister (2015) summarised the risk factors associated with the development of chronic pain postoperatively as being indicative of either peripheral or central neural sensitisation, neural trauma or adverse psychology. Considering this and the similar VAS scores within 7 days postoperatively, acute pain may result in a similar incidence and intensity as chronic pain. Second, depression and anxiety among patients that undergo neurosurgery are conditions associated with the development of chronic headache. The intensity of depression and anxiety has a positive correlation with the frequency of post-craniotomy headache (Rocha et al. 2008). Last, since the post-craniotomy headache involves the meninges, pericranial muscles and soft tissues, it is unlikely that scalp infiltration can prevent the onset of chronic headache.

According to previous research, the incidence and intensity of postoperative headache tends to decrease with the passage of time (Rocha-Filho 2015). Despite the low incidence of the chronic post-craniotomy pain, which tends to decrease with time, everyday life can be negatively influenced by such pain because of its long-lasting nature. As reported previously, neuropathic pain is probably the most common type of persistent postsurgical pain (Kehlet et al. 2006). Allodynia, hyperalgesia, and sensitisation have been reported in proximity to the surgical incision site several months postoperatively (Mosek et al. 1999). The neurons, glia, and immune cells all play a role in the development of chronic pain (Mifflin and Kerr 2014). The risk factors, for example, genetic susceptibility, preceding pain, psychosocial factors, bacterial infection, hypersensitivity, age, and sex, may contribute to persistent postoperative pain in unique ways (Kehlet et al. 2006). Preemptive, preventive, and multimodal analgesia are currently being practiced during the operative procedures, but whether they produce a clinically meaningful reduction in the intensity or duration of chronic pain remains unclear.

There are several limitations to our study. Given the limitations of VAS, we combined VAS and morphine consumption to assess postoperative pain to better reflect the effects of analgesia. As reported previously, questionnaires such as the Brief Pain Inventory and quantitative sensory testing (QST) have been validated for the assessment of chronic pain (Wilder-Smith et al. 2003). Neuroplasticity quantified by QST may be a significant factor in determining acute and perhaps chronic pain outcomes postoperatively (Woolf and Salter 2000). Furthermore, we may pay attention to neuropathic pain which affect the life of patients in future research.

The preoperative scalp infiltration with 0.5 % ropivacaine in patients undergoing craniotomy lessened the haemodynamic responses during scalp incision. Although there was no advantage of the scalp infiltration with 0.5 % ropivacaine in postoperative VAS, acute or chronic pain over placebo, the infiltration with ropivacaine did decrease morphine consumption during the first 24 h postoperatively and prolonged the time to first rescue analgesic requirement postoperatively.
